# Seeing is believing: observation of migrasomes

**DOI:** 10.52601/bpr.2023.230024

**Published:** 2024-04-30

**Authors:** Yuwei Huang, Li Yu

**Affiliations:** 1 School of Basic Medical Sciences, Translational Medicine Institute, Xi’an Jiaotong University, Xi’an 710049, China; 2 State Key Laboratory of Membrane Biology, Tsinghua University-Peking University Joint Center for Life Sciences, Beijing Frontier Research Center for Biological Structure, School of Life Sciences, Tsinghua University, Beijing 100084, China

**Keywords:** Migrasome, Labeling, Imaging, *In vitro* reconstitution

## Abstract

Migrasomes are a novel type of cell organelle that form on the retraction fibers at the rear of migrating cells. In recent years, numerous studies have unveiled the mechanisms of migrasome formation and have highlighted significant roles of migrasomes in both physiological and pathological processes. Building upon the strategies outlined in published works and our own research experiences, we have compiled a comprehensive set of protocols for observing migrasomes. These step-by-step instructions encompass various aspects such as cell culture, labeling, imaging, *in vitro* reconstitution, and statistical analysis. We believe that these protocols serve as a valuable resource for researchers exploring migrasome biology.

## INTRODUCTION

Migrasomes are newly identified organelles characterized as vesicular structures with a diameter ranging between 0.5–3 micrometers. They emerge at the intersection or tips of retraction fibers located at the rear of migrating cells. Comprising various biological molecules like proteins, nucleic acids, and lipids, migrasomes play a role in numerous physiological processes. Electron microscopy observations first recognized migrasomes in 2012, showcasing their distinct morphology with a thin tether and multiple small intraluminal vesicles, each approximately 50 nanometers in diameter. The discovery of Tetraspanin4 as the inaugural migrasome marker protein through candidate gene overexpression screening made it possible to study migrasomes using light microscopy (Ma *et al.* 2015).

Current research delineates three primary functions of migrasomes: they act as conduits for the release of signaling molecules, shaping the cellular micro-environment. Leveraging their unique spatiotemporal characteristics, migrasomes release the chemokine CXCL12, influencing zebrafish embryonic development by guiding DFC cells to the dorsal cavity, thus promoting regular organ development (Jiang *et al.* 2019). Likewise, in chicken embryos, monocytes instigate angiogenesis by discharging chemokine CXCL12 and vascular endothelial growth factor (VEGFa) via migrasomes, aiding capillary formation and arrangement (Zhang *et al.* 2022). In addition, migrasomes support horizontal material and information transfer between cells. For instance, cells can transfer *Pten* mRNA and PTEN protein to PTEN^-/-^ recipient cells through migrasomes, enabling the recipients to produce functional PTEN protein (Zhu *et al.* 2021). Finally, migrasomes allow cells to discard cellular "waste", such as malfunctioning mitochondria, ensuring mitochondrial equilibrium and upholding cellular vitality (Jiao *et al.* 2021).

Recent years have witnessed a growing number of studies on migrasomes in diseases, revealing their significant roles in pathological processes and proposing their potential applications in disease diagnosis and clinical treatment. Schmidt-Pogoda *et al*. discovered the presence of migrasomes in the brains of stroke patients (Schmidt-Pogoda *et al.* 2018), while Liu *et al*. observed an increased production of migrasomes in injured kidney podocytes compared to healthy ones. Liu *et al*. suggested that urinary podocyte migrasomes could serve as diagnostic markers for the early detection of podocyte injury (Liu *et al.* 2020). Additionally, Migrasomes are excessively generated by macrophage lineage cells upon amyloid protein stimulation, thereby contributing to complement-dependent blood vessel damage (Hu *et al.* 2023). Furthermore, it has been discovered that Migrasomes derived from Pigmented epithelium cells promote the progression of proliferative vitreoretinopathy (Wu *et al.* 2022).

The genesis of migrasomes is a biologically and biophysically driven process. Biologically, a cell decides whether to form migrasomes and even designates formation sites at the migration forefront. The formation of SMS2 foci on the cell substrate membrane is pivotal for migrasome formation. Locally synthesized SM furnishes crucial lipid materials for migrasome infrastructure (Liang *et al.* 2023). The PIP5Ka-PIP2-RAB35 axis transmits migrasome formation signals and modulates the Integrins' recruitment (Ding *et al.* 2023), which establishes anchoring points that precede migrasome formation (Wu *et al.* 2017). It is reported that a straight persistently or turning migration state orchestrates migrasome formation by shaping retraction fibers (Fan *et al.* 2022). PD-L1 was also reported to promote migrasome formation which suggests migrasome potential role in the immune checkpoint process (Wang *et al.* 2022). Biophysically, tetraspanin proteins and cholesterol-rich microdomains converge at these anchoring points, sensing the curvature (Dharan *et al.* 2022; Dharan *et al.* 2023) to assemble to generate a micrometer-sized macrodomain. This process enhances local membrane bending rigidity, culminating in migrasome creation (Huang *et al.* 2019).

Given their size and formation timeline, migrasomes are aptly suited for light microscopy observation. Subsequent research has identified various proteins and lipids, such as Tspans, Integrins, PH-domain, cholesterol, and sphingomyelin (SM), as migrasome biomarkers, enhancing their observability. An *in vitro* reconstitution approach, offering minimal factors and relatively quantitative characteristics, is invaluable for unraveling the intricacies of migrasomes. In this context, we present a basic protocol summary for migrasome observation, encompassing cell culture, migrasome labeling and reconstitution, microscopy-based migrasome imaging, and migrasome statistics.

## MATERIALS AND EQUIPMENT

### Reagents and supplies

• DMEM (HyClone, Cat. #SH30243.01)

• Penicillin-streptomycin solution (ThermoFisher Scientific, Cat. #15140122)

• SMM 293-TII Expression Medium (Sinobiological, Cat. #M293TII)

• GlutaMAX (ThermoFisher Scientific, Cat. #35050061)

• Fetal bovine serum (Biological Industries, Cat. #04-001-1ACS)

• Fetal bovine serum (Vivicum, Cat. #9906-85)

• Vigofect (Vigorous, Cat. #T001)

• Lipofectamine^TM^ 3000 transfection (Invitrogen, Cat. #L3000015)

• Streptavidin (Sigma-Aldrich, Cat. #85878)

• BSA (Bovine Serum Albumin) (Amresco, Cat. #0332)

• BSA-Biotin (Solarbio, Cat. #SHB263)

• Fos-Choline-12 (Anatrace, Cat. #F308)

• BIO-BEADS SM-2, BT, 20-50 (Bio-Rad, Cat. #1528920)

• POPC (Avanti Polar Lipids, Cat. #850457)

• DOPE (Avanti Polar Lipids, Cat. #850725)

• POPS (Avanti Polar Lipids, Cat. #840034)

• 16:0 Biotinyl Cap PE (Avanti Polar Lipids, Cat. #870277)

• Liss-Rhodamine-PE (Avanti Polar Lipids, Cat. #810150)

• Cholesterol (Sigma-Aldrich, Cat. #C8667)

• d-Desthiobiotin (Sigma-Aldrich, Cat. #D1411)

• Strep-Tactin^®^ Sepharose^®^ 50% suspension (IBA, Cat. #2-1201-010)

• Fibronectin (Invitrogen, Cat. #PHE023)

• FilipinⅢ (Biogems, Cat. #4804999)

• Anti-ITGA5 antibody (BD Biosciences, Cat. #553319)

• Anti-ITGB1 antibody (BD Biosciences, Cat. #553715)

• Donkey anti-Rat IgG (H+L) Highly Cross-Adsorbed Secondary Antibody, Alexa Fluor^TM^ 647 (Invitrogen, Cat. #A78947)

• Wheat germ agglutinin (WGA) with Alexa Fluor488 (Invitrogen, Cat. #W11261)

• Wheat germ agglutinin (WGA) with Alexa Fluor555 (Invitrogen, Cat. #W32464)

• Wheat germ agglutinin (WGA) with Alexa Fluor647 (Invitrogen, Cat. #W32466)

• Wheat germ agglutinin (WGA) with Alexa Fluor350 (Invitrogen, Cat. #W11263)

• FM™ 4-64FX (Invitrogen, Cat. #F34653)

• Calcein-AM (Invitrogen, Cat. #C3099)

• Glass slides (Fisher Scientific, Cat. #12-544-14)

• Standard Wall Borosilicate Tubing (WPI, Cat. #1B100-6)

• Confocal dishes (In Vitro Scientific, Cat. #D35-20-1-N)

### Experimental models: cell lines

• NRK: ATCC (ATCC^®^ Number: CRL-6509^TM^)

• NCTC clone 929 (L929): ATCC (ATCC^®^ Number: CCL-1^TM^)

• 293F: Laboratory of Yigong Shi, Tsinghua University

• MGC803: Laboratory of Zhijie Chang, Tsinghua University

### Software and algorithms

• Image J: National Institutes of Health, N/A

• GraphPad Prism: GraphPad Software, GraphPad Prism5 or 7

• Nikon analysis: Nikon, NIS-elements-AR5.0

### Equipment

• Vesicle Prep-Pro machine: Nanion, Vesicle Prep Pro

• Nucleofector: Lonza, Amaxa Nucleofector Ⅱ Device

• Plasma cleanser: Harrick Plasma, PDC-32G

• Confocal microscope: Olympus, Fluoview 3000

• NIKON A1 microscope: NIKON, NIKON A1

## STEP-BY-STEP PROCEDURE

### Step 1: Cell culture for migrasome observation

Migrasome is a migration-dependent organelle. Adequate space and an appropriate substrate for cell migration are essential for a cell to produce migrasomes. With a lifespan of around 400 minutes, the migrasome's observation time window is crucial for achieving high-quality visualization of both migrasomes and retraction fibers. In this context, we present our fundamental cell culture protocol aimed at determining whether a cell can produce migrasomes and ensure optimal migrasome imaging. If you opt for a cell type not previously associated with migrasome studies, it's recommended to adapt the protocol provided, paying particular attention to cell seeding concentration and the observation time window, which largely depends on your cell's proliferation rate.

#### Step 1.1: Choose the proper extra cellular matrix [TIMING 3 d]

According to Wu *et al*., the correct pairing of integrins with the corresponding extracellular matrix (ECM) determines migrasome formation (Wu *et al.* 2017). To monitor this formation, it's crucial first to choose the right ECM that matches the most highly expressed Integrin subtype in a specific cell type.

Step 1.1.1: Determine the expression level of each Integrin in the specific cell line using online databases, such as www.proteinatlas.com, to identify the most highly expressed Integrin subtype.

Step 1.1.2: Use quantitative real-time PCR to validate the findings from the first step with your own cells and confirm the predominant Integrin subtype.

Step 1.1.3: Based on the correlation between Integrins and the ECM to select the most suitable ECM for your cells.

#### Step 1.2: Confocal dish preparation [TIMING within 2 h]

Step 1.2.1: Make sure that your cells are in a healthy state, especially without contamination.

Step 1.2.2: Pre-coat the glass bottom confocal dish with proper ECM, for example, fibronectin (10 μg/mL in PBS) for 30 min to 2 h at 37 °C.

**[TIP]** Please pay attention to make sure the ECM liquid covers all the glass bottoms and avoid local desiccation.

Step 1.2.3: Wipe out the ECM liquid and add the cell culture medium immediately to keep the glass bottom in the wet state.

#### Step 1.3: Cell seeding [TIMING 16 h]

Step 1.3.1: Prepare cell suspension and then seed 0.8–2 × 10^4^ cells onto an ECM pre-coated single-well glass bottom confocal dish to ensure sufficient space for cell migration.

**[TIP]** The cell density is highly related to the cell proliferation speed, the principle for cell seeding density is to ensure the cell confluency reaches 30%–40%, 15 h after seeding.

Step 1.3.2: Culture cells in an incubator supplemented with 5% CO_2_ for 15 h at 37 °C.

### Step 2: Migrasome labeling

Migrasomes are single-membrane vesicular structures. They can be identified by labeling either the migrasome membrane or the migrasome cytosol. Based on previous studies, several proteins and lipids are known to localize to or even be enriched in migrasomes, offering numerous targets for migrasome labeling. We outline three methods: labeling migrasomes using marker proteins tagged with fluorescent proteins, using fluorescence visualization dyes, and using fluorescence-conjugated antibodies. We have summarized the advantages and disadvantages of these three methods in Table 1. Depending on your research objectives and available experimental materials, you can choose one or several methods from the following established protocols.

**Table 1:** Comparison of methods for migrasome labeling

Name of methods	Marker proteins tagged with fluorescent proteins	Fluorescence visualization dyes	Fluorescence-conjugated antibodies
Sample types	Living cell	Living cell or fixed cell	Fixed cell
Advantages	Suitable for long-term time-lapse imaging	Easy and fast	Display the basal state of migraosmes and the endogenous state of targets
Disadvantages	Over-expression of marker proteins may have impact on migrasome formation	Imaging quality is highly related to endocytosis,toxicity and photobleaching rate of the dye	Tedious steps and not able to real-time observation
Key restrictive factor	Efficiency of gene transfection and protein expression	Abundance of target to the dye in specific cell type	Specificity of antibodies

#### Step 2.1: Label migrasomes by expression of marker proteins [TIMING 1 d]

According to the previous studies, a list of migrasome marker proteins, such as Tetraspanin4, Integrinα5, PH domain and so on (Table 2), when fused with a fluorescent protein tag, could clearly label migrasomes when expressed in a cell.

**Table 2:** Summary of migrasome protein markers

Protein biomarkers of migrasome	Role in migrasome formation	Existing data evidences
TSPAN 1	Assebbly to Tetraspanin-enriched microdomains to drive migrasome formation	Imaging data (Huang *et al.* 2019)
TSPAN 2		Imaging data (Huang *et al.* 2019)
TSPAN 4		Imaging and Biochemical data (Ding *et al.* 2023; Huang *et al.* 2019; Jiang *et al.* 2019; Jiao *et al.* 2021; Liang *et al.* 2023; Ma *et al.* 2015; Wu *et al.* 2017*,* 2022; Zhang *et al.* 2022; Zhao *et al.* 2019)
TSPAN 9		Imaging data (Jiao *et al.* 2021)
CD81		Imaging data (Huang *et al.* 2019)
CD82		Imaging data (Huang *et al.* 2019)
ITGA5	Anchor migraosmes to the extra cellular matrix	Imaging and biochemical data (Ding *et al.* 2023; Jiang *et al.* 2019; Jiao *et al.* 2021; Wu *et al.* 2017; Zhao *et al.* 2019)
ITGB1		Imaging data and biochemical data (Jiang *et al.* 2019; Wu *et al.* 2017)
SMS2	SMS2 foci determine sites for migrasome formation	Imaging data (Liang *et al.* 2023)
PLCδ-PH-domain (probe for PI(4,5)P2)	Phosphatidylinositol (4,5)-bisphosphate-Rab35 axis regulates migrasome formation	Imaging data (Ding *et al.* 2023; Jiang *et al.* 2019; Wu *et al.* 2021)
RAB35		Imaging data (Ding *et al.* 2023)
PIP5K1A		Imaging data (Ding *et al.* 2023)
NDST1	Unknown	Imaging and biochemical data (Zhang *et al.* 2022; Zhao *et al.* 2019)
PIGK		Imaging and biochemical data (Zhao *et al.* 2019)
CPQ		Imaging and biochemical data (Jiao *et al.* 2021; Zhang *et al.* 2022; Zhao *et al.* 2019)
EOGT		Imaging and biochemical data (Zhao *et al.* 2019)

##### Step 2.1.1: Cell transfection by chemical reagents

By use of Vigofect.

Step 2.1.1.1: Take a 35-mm cell culture dish of cells as an example, when the cells reach a confluence of 60% to 80% with good state, replace 2 mL of fresh complete medium 1 hour before transfection.

Step 2.1.1.2: Take 5 μg of endotoxin-free high-purity plasmids and dilute it in 100 μL of PBS. Mix well and leave at room temperature.

Step 2.1.1.3: In another 1.5 mL EP tube, add 100 μL of PBS, then slowly add 2 μL of VigoFect Transfection Reagent and mix well. Leave at room temperature for 5 min.

Step 2.1.1.4: Drop-wise add the diluted VigoFect Transfection Reagent to the diluted plasmid solution. Mix well and leave at room temperature for 15 min.

Step 2.1.1.5: Drop-wise add the prepared transfection mixture from Step 2.1.1.4 to the cells prepared in Step 2.1.1.1. Gently shake the dish to mix the cells and incubate them in a 37 °C cell culture incubator with 5% CO_2_.

Step 2.1.1.6: After 6 h of transfection, remove the medium containing the transfection reagent and replace it with a fresh complete medium to reduce the cytotoxic effects of VigoFect reagent on cells.

Step 2.1.1.7: After 12 h of transfection, the cells can be washed with PBS and trypsinized, then seeding.

##### Step 2.1.2: By use of Lipofectamine3000

Step 2.1.2.1: Take a 35-mm cell culture dish of cells as an example, when the cells reach a confluence of 80%–90% with a good state. Dilute 5 μg of plasmids in 125 μL of PBS, then add 10 μL of P3000, mix well.

Step 2.1.2.2: In another 1.5 mL EP tube with 125 μL of PBS, add 7.5 μL of Lipofactamine3000 Reagent and mix well.

Step 2.1.2.3: Drop-wise add the diluted plasmid solution to Lipofactamine3000 Transfection Reagent. Mix well and leave at room temperature for 5 min.

Step 2.1.2.4: Drop-wise add the prepared transfection mixture to the cells. Gently shake the dish to mix the cells and incubate them in a 37 °C cell culture incubator with 5% CO_2_.

Step 2.1.2.5: After 12 h of transfection, the cells can be washed with PBS and trypsinized, then seeding.

##### Step 2.1.3 Cell transfection by electroporation transfection

For cells proper to use electroporation transfection, such as Norway rat kidney (NRK) cell, we recommend the following protocol for you. Here we use the Amaxa Nucleofector system from Lonza, Germany, for electroporation transfection.

Step 2.1.3.1: Taking a 6-cm cell culture dish of NRK cells as an example, when the cells reach a confluence of approximately 90% and are in good condition, perform PBS washing and trypsin digestion to prepare cell suspension.

Step 2.1.3.2: After pipetting and thoroughly mixing the cells, aspirate 1/3 of the cell suspension and transfer it to a 1.5 mL EP tube. Centrifuge at 600 *g* for 5 min.

Step 2.1.3.3: Remove the culture medium and add 100 μL of cell electroporation buffer (with 3 μg of endotoxin-free high-purity plasmids) to re-suspend the cells.

Step 2.1.3.4: Gently mix and transfer the mixture to a 0.2 cm electroporation cuvette, taking care to avoid bubble formation during the process to improve electroporation efficiency.

Step 2.1.3.5: Place the electroporation cuvette in the Amaxa Nucleofector system and select the corresponding electroporation program for NRK cells.

Step 2.1.3.6: After electroporation, add 1 mL of fresh culture medium to the electroporation cuvette to evenly re-suspend the cells.

Step 2.1.3.7: Take an appropriate amount of cells and plate them in an ECM coated confocal dish. Thoroughly mix and place the dish in a 37 °C cell culture incubator with 5% CO_2_ for 15 h.

#### Step 2.2: Label migrasomes by immuno-fluorescence staining [TIMING 0.5 d]

##### Step 2.2.1: Cell sample fixation

Step 2.2.1.1: Instead of small cover slips, we recommend seed cells on ECM-coated glass bottom confocal dishes for immunofluorescence staining of samples to avoid destroying the structure of migrasomes and retraction fibers.

Step 2.2.1.2: Remove the culture medium and add fixative Solution A (mixed with equal volumes of 4% paraformaldehyde and regular culture medium, preheated to 37 °C) for 5min.

Step 2.2.1.3: Remove fixative solution A and add fixative solution B (4% paraformaldehyde preheated to 37 °C) to fix samples at room temperature for 10 min.

##### Step 2.2.2 Immuno-fluorescence staining

Step 2.2.2.1: Gently wash the samples three times with PBS for 5 min each time.

Step 2.2.2.2: Add permeabilization solution (PBS solution containing 0.05% saponin and 10% FBS) at room temperature for 5 min.

Step 2.2.2.3: After removing the permeabilization solution, add blocking solution (PBS solution containing 10% FBS) to the cell sample and incubate at room temperature for 30 min.

Step 2.2.2.4: Remove the blocking solution, add diluted primary antibody (diluted in blocking solution at working concentration), and incubate at room temperature for 1 h.

Step 2.2.2.5: Remove the diluted primary antibody and wash three times with PBS for 10 min each time.

Step 2.2.2.6: Add the desired dilution of fluorescent secondary antibody (usually diluted at 1:500 in blocking solution) and incubate at room temperature in the dark for 1 h.

Step 2.2.2.7: Remove the fluorescent secondary antibody and wash three times with PBS for 10 min each time.

Step 2.2.2.8: Due to the inability to cover slip, samples prepared in laser confocal specialized dishes cannot be stored for a long time and should be imaged as soon as possible.

**[TIP]** If the samples cannot be imaged immediately, the laser confocal dishes need to be sealed with sealing film and covered with aluminum foil to avoid light exposure. They can be stored in a refrigerator at 4 °C for several days.

**Figure 1:**
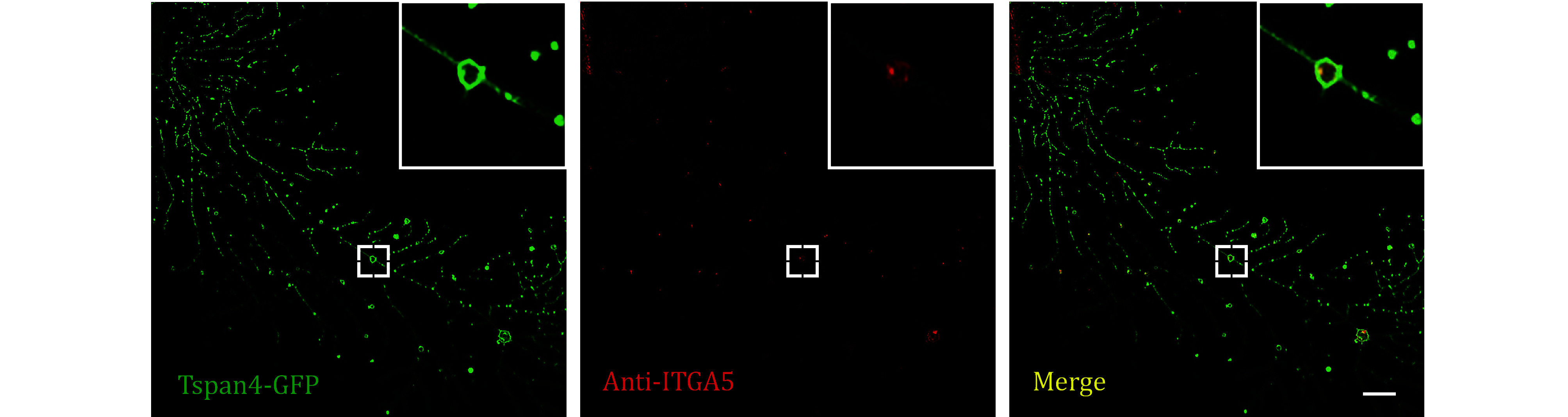
Images of Tetraspanin4-GFP over-expression L929 cell immuno-fluorescence stained by anti-ITGA5 antibodies were collected by confocal microscopy. Green, GFP; red, Alexa fluor-647; yellow, merge. Scale bar, 10 μm

**Figure 2:**
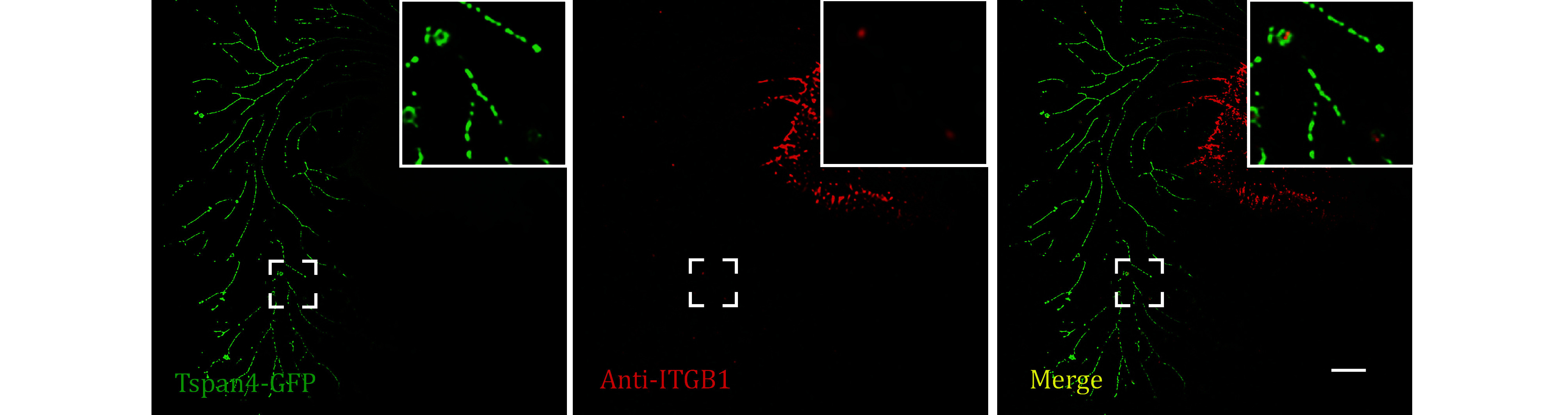
Images of Tetraspanin4-GFP over-expression L929 cell immuno-fluorescence stained by anti-ITGB1 antibodies were collected by confocal microscopy. Green, GFP; red, Alexa fluor-647; yellow, merge. Scale bar, 10 μm

**[TIP]** The representative images were displayed in Figs. 1 and 2.

#### Step 2.3: Label migrasomes by dyes [TIMING within 2 h]

##### Step 2.3.1 WGA staining

Step 2.3.1.1: Fluorescence-conjugated WGA could be purchased from Thermo Fisher Scientific. WGAs usually were stocked in PBS with a concentration of 1 mg/mL at –20 °C.

(A) For living-cells

Step 2.3.1.2: Dilute the fluorescence-conjugated WGA to 1 μg/mL in a fresh preheated culture medium.

Step 2.3.1.3: Discard the old culture medium in the cell sample, add 500 μL of freshly prepared diluted WGA solution to the glass bottom area of the culture dish, and incubate at 37 °C for 5–15 min.

Step 2.3.1.4: Discard the WGA solution, add 2 mL of fresh preheated culture medium at 37 °C and quickly observe.

**[TIP]** If you need to take time-lapse images, please keep the WGA molecules in the cell culture system to maintain the supplementary enough WGA molecules for migrasome labeling.

(B) For fixed cells

Step 2.3.1.5: Remove the PBS solution and add 1 μg/mL WGA diluted in PBS at room temperature for 10 min.

Step 2.3.1.6: Remove the staining solution and wash with PBS once.

**Figure 3:**
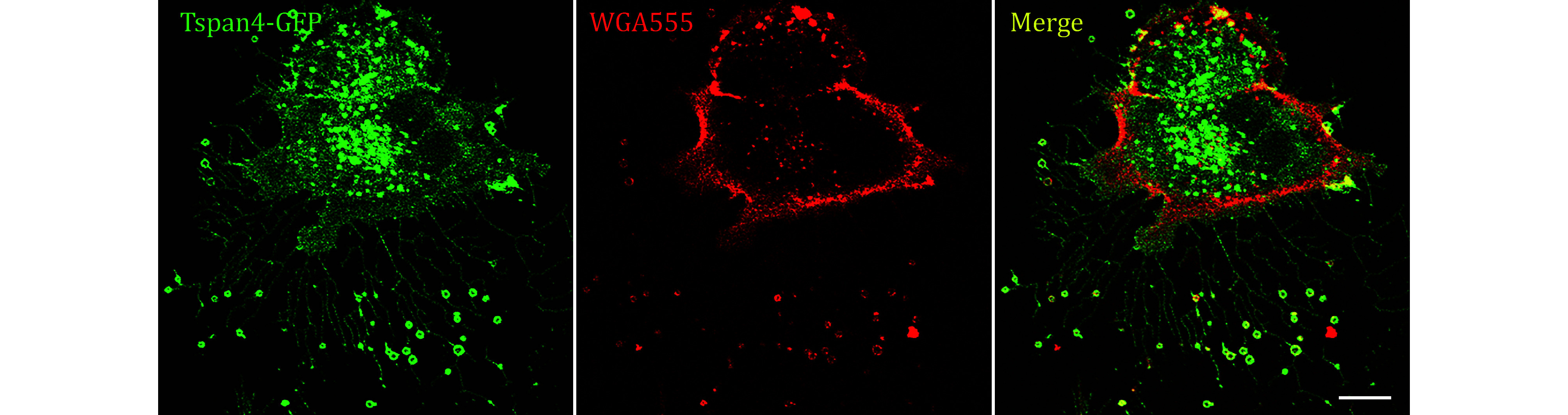
Images of Tetraspanin4-GFP over-expression NRK cells stained by WGA555 were collected by confocal microscopy. Green, GFP; red, WGA555; yellow, merge. Scale bar, 10 μm

**[TIP]** The representative images are displayed in Fig. 3.

##### Step 2.3.2: FM4-64 staining

Step 2.3.2.1: FM4-64 was dissolved in water to 5 mg/mL concentration to be stocked at –20 °C.

(A) For living-cells

Step 2.3.2.2: Dilute the FM4-64 stocking solution to 5 μg/mL in the fresh preheated culture medium.

Step 2.3.2.3: Remove the old culture medium in the cell sample, add 500 μL of freshly prepared diluted FM4-64 solution to the glass bottom area of the culture dish, and incubate at 37 °C for 15 min.

(B) For fixed cells

Step 2.3.2.4: Remove the PBS solution and add 5 μg/mL FM4-64 diluted in PBS at room temperature for 15 min.

Step 2.3.2.5: Remove the staining solution and wash with PBS once.

**Figure 4:**
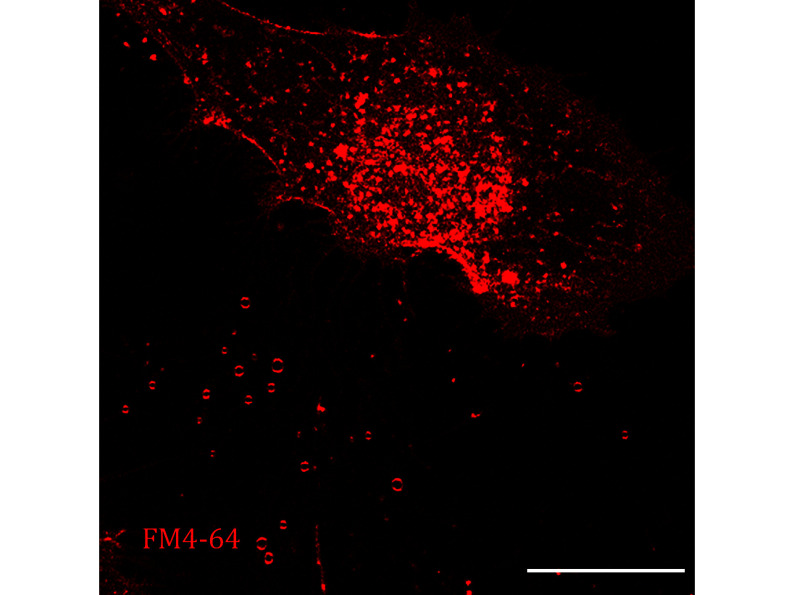
Images of Tetraspanin4-GFP over-expression NRK cells stained by FM4-64 were collected by confocal microscopy. Scale bar, 10 μm

**[TIP]** The representative images are displayed in Fig. 4.

##### Step 2.3.3: Calcein staining

Step 2.3.3.1: Dilute the Calcein-AM (stocked in DMSO with the concentration of 2 mmol/L) to 50 μmol/L in the fresh preheated culture medium.

Step 2.3.3.2: Remove the old culture medium in the cell sample and add 500 μL of freshly prepared diluted Calcein-AM solution to the glass bottom area of the culture dish, and incubate at 37 °C for 10 min.

Step 2.3.3.3: Discard the Calcein-AM staining solution and add 2 mL of fresh preheated culture medium at 37 °C.

**[TIP]** Calcein is suitable for living cell staining.

**Figure 5:**
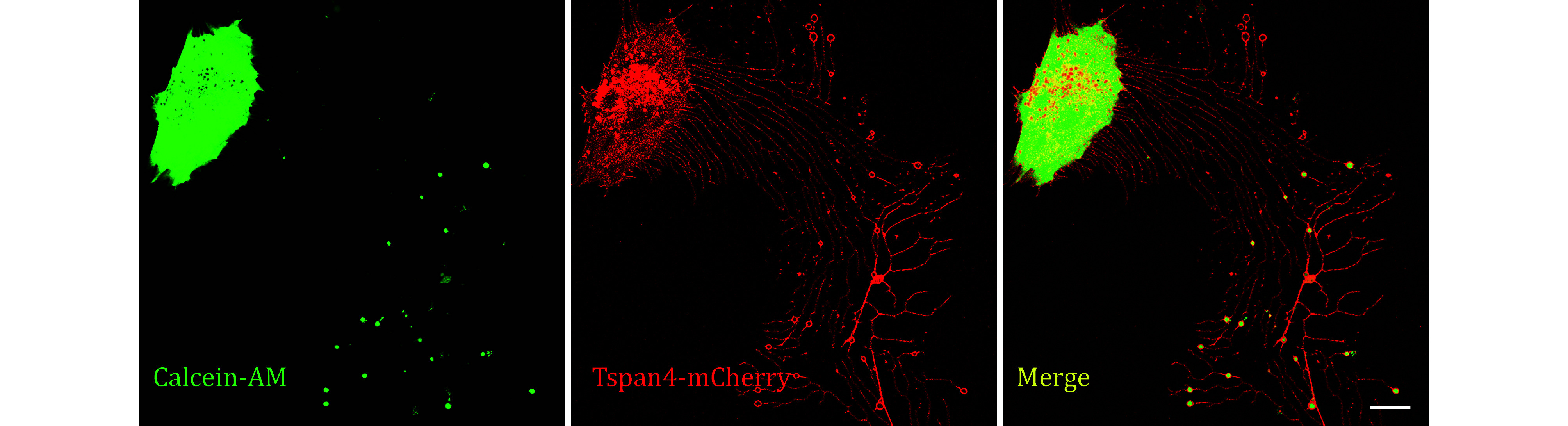
Images of Tetraspanin4-mCherry over-expression L929 cells stained by Calcein-AM were collected by confocal microscopy. Green, Calcein-AM; red, mCherry; yellow, merge. Scale bar, 10 μm

**[TIP]** The representative images are displayed in Fig. 5.

##### Step 2.3.4: Filipin III staining

Step 2.3.4.1: Prepare the fixed cell samples as described before.

Step 2.3.4.2: Freshly dissolve the Filipin III solid in DMSO to 2 mg/mL and avoid lights.

Step 2.3.4.3: Discard the PBS solution on fixed cells and add 2 μg/mL Filipin III working solution diluted in PBS to stain the samples at 37 °C for 15 min to 1 h.

Step 2.3.4.4: Discard the staining solution and add 2 mL of PBS to quickly observe and photograph the cells under a laser scanning confocal microscope.

**[TIP]** Due to the easy quenching of Filipin III fluorescence, it is recommended to lower the laser intensity and shorten the exposure time when observing and photographing.

**[TIP]** Fillipin III is proper for fixed-cell samples

**Figure 6:**
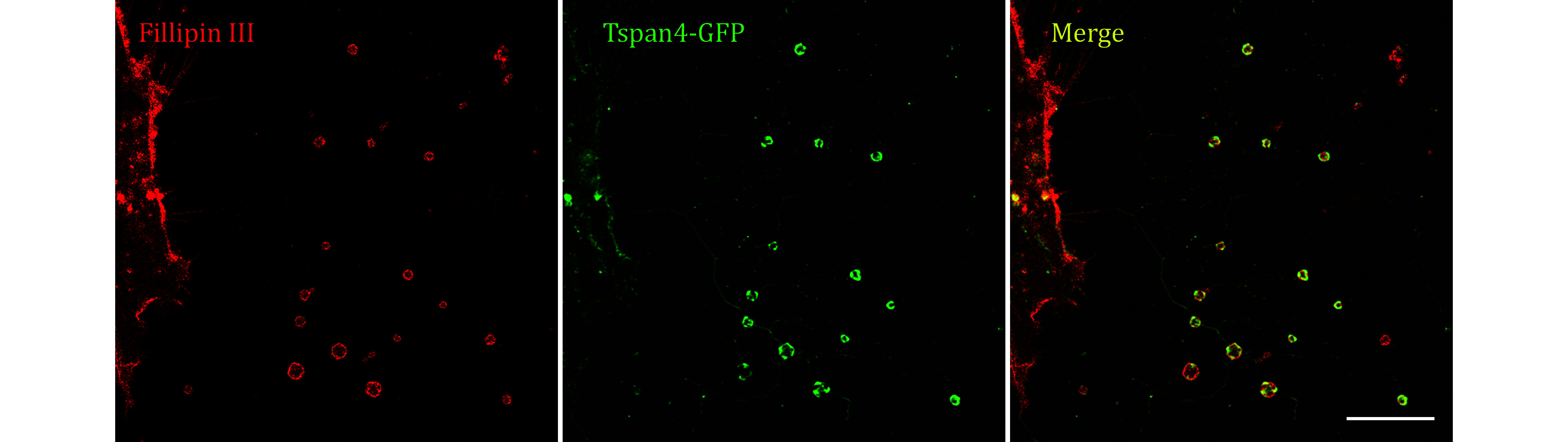
Images of Tetraspanin4-GFP over-expression NRK cell stained by Fillipin III were collected by confocal microscopy. Green, GFP; red, Fillipin III; yellow, merge. Scale bar, 10 μm

**[TIP]** The representative images are displayed in Fig. 6.

##### Step 2.3.5: NT-Lysenin staining

**[TIP]** There are no commercialized fluorescent proteins conjugated NT-Lysenin probes. You could purify the His-EGFP-NT-Lys or His-mCherry-NT-Lys as described previously (Liang *et al.* 2023).

(A) For living-cell imaging. Remove the cell medium and add preheated medium with 10 μg/mL NT-Lys at 37 °C for 2 h.

(B) For fixed cells. Remove the PBS solution and add 10 μg/mL NT-Lys in PBS at 37 °C for 15 min, then wash the sample with PBS once.

**Figure 7:**
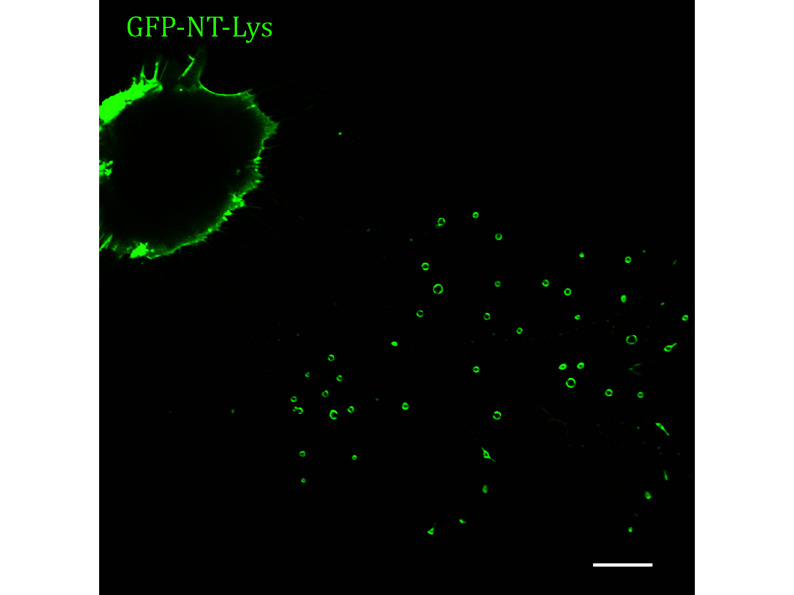
Image of L929 cells stained by GFP-NT-Lys was collected by confocal microscopy. Scale bar, 10 μm

**[TIP]** The representative images are displayed in Fig. 7.

Different dyes for migrasome labeling are listed in Table 3.

**Table 3:** Summary of different dyes for migrasome labeling

Category	Targets	Suitable sample types	Commer-cialization	Name of dyes	Ex/Em (nm)
Wheat Germ Agglutinin	N-acetylglucosamine and N-acetylneuraminic acid (sialic acid) residues	Living cell and fixed cell	Yes	WGA, Alexa Fluor 350 conjugates	346/442
				WGA, Alexa Fluor 488 conjugates	495/519
				WGA, Alexa Fluor 555 conjugates	555/565
				WGA, Alexa Fluor 594 conjugates	590/617
				WGA, Alexa Fluor 633 conjugates	632/647
				WGA, Alexa Fluor 647 conjugates	650/668
				WGA, Alexa Fluor 680 conjugates	679/702
FM dyes	Cell membrane	Living cell and fixed cell	Yes	FM4-64	515/640
				FM1-43	472/580
Calcein-AM	Living cell cytosol	Living cell	Yes	Calcein-AM, green version	494/517
				Calcein-AM,orange version	577/590
FillipinⅢ	Cholsterol	Fixed cell	Yes	FillipinⅢ	360/480
NT-Lysenin	Sphygomyelin	Living cell and fixed cell	No	EGFP -NT-Lys	488/507
				mChery-NT-Lys	587/610

#### Step 3: Migrasome *in vitro* reconstitution

*In vitro* reconstitution provides a relatively controlled and quantitative system to further decipher the mechanisms of migrasome formation. This approach serves as a powerful complement to *in vivo* systems. We have developed a series of methods to reconstitute migrasomes, and these systems have successfully helped to unveil the roles of Tetraspanin proteins and cholesterol in migrasome formation (Huang *et al.* 2019). Here, we outline detailed protocols, from the purification of Tetraspanin proteins to the preparation of protein-embedded bio-membranes, and finally, to migrasome reconstitution.

##### Step 3.1: Tetraspanin protein purification [TIMING 5 d]

The TSPAN4 protein used in this study was expressed and purified using 293F cells. The specific procedures are as follows:

Step 3.1.1: 293F cells were suspended in SMM 293-TII medium containing 10% penicillin-streptomycin without serum and cultured at 37 °C with 8% CO_2_ and 110 r/min. The cell density for passaging was maintained at 2–3 × 10^6^ cell/mL, and the seeding density for passaging was 0.3–0.5 × 10^6^ cell/mL.

Step 3.1.2: When the cell density reached 0.8–1.2 × 10^6^ cell/mL, plasmid transfection was performed (based on 1 L of cells). Firstly, 5 mL of PEI (stock concentration of 1 mg/mL) was added to 45 mL of medium, and mixed well, and then 2 mg of strep-TSPAN4-GFP plasmids was added and mixed again. After 15 min of incubation, this mixture was added to the cell suspension and cultured for an additional 3–4 days to achieve protein expression.

Step 3.1.3: The cell suspension was transferred to a 50 mL centrifuge tube and centrifuged at 550 *g* for 5 min at 4 °C. The pellet was washed with PBS, centrifuged again, and the supernatant was discarded.

**[TIP]** If protein extraction was not urgent, the cell pellet could be frozen rapidly in liquid nitrogen and stored at –80 °C.

Step 3.1.4: The cell pellet was resuspended in 5 mL of 4 °C Tris-HCl buffer (with protease inhibitors).

Step 3.1.5: The suspension was transferred to a Dounce homogenizer and homogenized on ice for approximately 100 strokes to promote cell lysis (cell rupture could be detected using Trypan blue staining). The homogenate was then centrifuged at 30,000 *g* for 1 h at 4 °C, and the supernatant was discarded.

Step 3.1.6: The pellet was resuspended in 10 mL of Tris-HCl buffer and mixed immediately with 2.5 mL of 10% Fos-Choline-12 (protease inhibitors should be added). The tube was incubated on a rotator at 4 °C for 2 h to extract the protein.

Step 3.1.7: The mixture was centrifuged at 30,000 *g* for 30 min at 4 °C.

Step 3.1.8: The supernatant from the previous step was transferred to an affinity chromatography column (previously washed with Tris-HCl buffer containing strep-tactin beads), sealed, confirmed with sealing film, and incubated on a rotator at 4 °C for 1 h.

Step 3.1.9: After incubation, when the supernatant had drained, the beads were washed with approximately 10 mL of washing buffer (Tris-NaCl buffer with 0.1% (*w*/*v*) Fos-Choline-12).

Step 3.1.10: The beads were incubated with 5 mL of elution buffer (Tris-NaCl buffer with 0.1% (*w*/*v*) Fos-Choline-12 and 10 mmol/L desthiobiotin) for a few minutes until the protein was dissolved in the liquid. The eluate was collected, and the beads were washed with 1 mL of elution buffer three times. The collected liquids were combined.

Step 3.1.11: SDS-PAGE gel electrophoresis and Coomassie brilliant blue staining were performed to preliminarily identify the purified protein in each step.

Step 3.1.12: The eluate was concentrated using a 30 kDa ultrafiltration centrifuge tube at 3,200 *g* for no more than 1 h at 4 °C, resulting in a final volume of less than 1 mL. The concentrated liquid was transferred to an EP tube.

Step 3.1.13: The protein concentration was measured, divided into aliquots, and rapidly frozen in liquid nitrogen.

**[TIP]** For proteins intended for long-term storage and proteo-liposome preparation, the protein concentration should be higher than 1 mg/mL.

**[TIP]** Western blotting (WB) can be considered for further verification of protein purity.

##### Step 3.2: Proteo-liposome preparation

###### Step 3.2.1: Small uni-laminer vesicles (SUVs) preparation [TIMING 0.5 d]

Step 3.2.1.1: Clean the glass bottle and micropipette used to make liposomes using a mixture of methanol and chloroform (*v*:*v* = 2:1). Repeat this step three times and then dry with N_2_ gas.

Step 3.2.1.2: Based on the desired lipid composition and ratio, transfer the appropriate lipid components into the glass bottle using a micropipette. Different samples may require 3–4 washes with chloroform. The lipid mass ratio in the formulation is DOPC∶DOPE∶POPS∶Cholesterol = 4∶2.5∶2.5∶1, and further with 1% biotin-PE and 0.1% Rhodamine-PE.

Step 3.2.1.3: Use N_2_ gas at an appropriate flow rate to evenly spread the mixed lipids as a thin film on the inner wall of the glass tube while rotating.

Step 3.2.1.4: To ensure complete solvent evaporation, leave the glass bottle open and dry in a 37 °C oven for 1 h.

Step 3.2.1.5: Add 100 μL of SUV buffer (usually determined by the final reaction system containing the liposomes) to the sealed glass bottle (sealed with parafilm) and shake on a 37 °C shaker for 1 h to ensure complete hydration of the lipids.

Step 3.2.1.6: Transfer the hydrated solution to an EP tube. Freeze and thaw the solution between liquid nitrogen (1 min) and a 42 °C water bath (3 min) for 12 cycles. The liquid will gradually become clear.

Step 3.2.1.7: Assemble the liposome extruder with a 1-μm pore size filter membrane and filter the freeze-thawed product 21 times.

**[TIP]** It is recommended to replace the filter membrane, needle, and accessories with MilliQ H_2_O wash three times for each sample.

Step 3.2.1.8: Transfer the filtered product (with sample and outlet needles on opposite sides, not on the same side) to a new EP tube, and the SUVs were freshly prepared.

###### Step 3.2.2: Insertion of Tspan4-GFP proteins onto SUVs [TIMING 1 d]

Step 3.2.2.1: Add an appropriate amount of 10% Fos-Choline-12 to the freshly prepared SUVs to reach the final detergent concentration of 0.1%, mix rapidly and rotate at 4 °C for 30 min.

Step 3.2.2.2: Add 10 μg of protein to the system, and add an equal volume of buffer solution to the control group. Rotate at 4 °C for 1 h.

Step 3.2.2.3: Weigh four portions of bio-beads, each portion weighing 80–120 mg.

Step 3.2.2.4: Add the first three portions to the mixture at intervals of 1 h, and let them adsorb overnight after the third addition. The adsorption process is performed on a rotator at 4 °C with 35 r/min the first twice addition, and with 25 r/min overnight.

Step 3.2.2.5: Transfer the liquid part to an EP tube containing the fourth portion of bio-beads and rotate at 4 °C for 1 h.

Step 3.2.2.6: The resulting liquid part is the final proteo-liposome product. It can be aliquoted according to experimental needs and stored at –80 °C after rapid freezing in liquid nitrogen.

##### Step 3.3: Proteo-GUV preparation

###### Step 3.3.1: By Electroformation methods [TIMING 0.5 d]

**[TIP]** GUVs with embedded proteins were prepared from protein-containing SUVs using the Vesicle Prep-Pro machine (Nanion).

Step 3.3.1.1: Wash the ITO-covered glass slides with dish detergent with the sponge and then rinse all the detergent off with tap-water.

Step 3.3.1.2: The slides need to be washed with pure methyl alcohol three times.

Step 3.3.1.3: Wash out methyl alcohol by running ddH_2_O three times, for the final wash, use MilliQ H_2_O.

Step 3.3.1.4: Dry the slides with N_2_ gas.

Step 3.3.1.5: Use a multimeter to confirm the ITO-covered glass side.

Step 3.3.1.6: A 20-µL drop of SUVs or proteo-liposomes was coated onto ITO-covered glass slides to form a lipid-protein film and dried for 30 min at 37 °C, avoiding light.

Step 3.3.1.7: The slides were placed into the Vesicle Prep-Pro machine and 270 µL of 300 mmol/L sorbitol buffer was applied to the slides.

Step 3.3.1.8: Electroformation was then carried out at 0.24 V and 9.9 Hz for 90 min at 37 °C.

Step 3.3.1.9: The GUVs were harvested after cooling down to room temperature and stored at 4 °C to be used within three days.

###### Step 3.3.2: By Gel-assisted hydration methods [TIMING 0.5 d]

Step 3.3.2.1: Prepare a 1% (*w*/*v*) agarose (low melting point) solution (10 mL is sufficient) by heating in a microwave on high until boiling. Mix well to ensure the agarose is completely dissolved. Clean the cover glass with glass-specific detergent, and for the final wash, use MilliQ H_2_O.

Step 3.3.2.2: Before coating, treat the cover glass with Plasma cleanser machinery for 1 min.

**[TIP]** This step helps to ensure uniform coating of the agarose on the cover glass, so the treated cover glass should be used as soon as possible.

Step 3.3.2.3: Add 200 μL of the hot agarose solution onto the surface of the cover glass, making sure the liquid fully covers the glass. Use fine-tipped forceps to hold one corner of the glass and allow one edge of the glass to touch the surface of a lint-free Kimwipe to remove excess liquid, ensuring that only a thin, uniform layer of agarose covers the glass surface.

Step 3.3.2.4: Place the treated cover glass in a 65 °C oven for 30 min to fully dry the surface. After removal, let it cool down and store it for later use.

**[TIP]** Prepared glass should be used as soon as possible and should not be stored at 4 °C for more than one week.

Step 3.3.2.5: Place the prepared cover glass, with the agarose-coated side facing up, in the bottom groove of the cover glass holder. Using a micropipette, dot the proteo-liposome mixture (25 μL) onto the glass surface, creating an array of small droplets, approximately 25–30 dots.

Step 3.3.2.6: Using N_2_ gas, dry the droplets as soon as possible and then add 200 μL of GUV physiological growth buffer (GUV buffer) to cover the glass surface.

**[TIP]** This step should be done gently to prevent liquid from overflowing the glass.

Step 3.3.2.7: Cover the glass with the matching cover of the holder and incubate in a 37 °C incubator for 30 min.

Step 3.3.2.8: After incubation, gently tap the side of the cover glass holder to encourage the generated GUVs to detach from the agarose. The collected liquid is then prepared GUVs, which should be used on the same day as they are prepared.

##### Step 3.4: Migrasome in-vitro reconstitution

###### Step 3.4.1: Migrasome reconstitution by flow channel [TIMING 3 d]

(A) Glass slips cleaning

Step 3.4.1.1: Place rectangle cover slips in a 50 mL tube and wash it alternately with MilliQ H_2_O and ethanol for 2–3 days. Finally, store it in MilliQ water. Before use, rinse with ethanol, and dry with N_2_ gas.

Step 3.4.1.2: Hydrophilic treatment by use of Plasma cleanser machinery. Vacuum the instrument for 30 s and then set it to a high level for 10 min of plasma discharge.

(B) Prepare the channels

Step 3.4.1.3: Use a cutter to cut double-sided adhesive tape (super adhesive type) into strips approximately 2 cm in width and 5 cm in length.

Step 3.4.1.4: Use forceps to attach the tape to the plasma-treated side of the cover glass, approximately 6–8 strips.

Step 3.4.1.5: Vertically affix the cross-shaped cover glass onto a tissue culture slide for physiological use, pressing firmly to ensure no overlapping channels.

(C) Reconstitution by flow

Step 3.4.1.6: Incubate the channel at room temperature with 20 μL of the blocking solution (10% BSA:1% BSA-biotin in HEPES buffer (10 mmol/L HEPES and 150 mmol/L NaCl, pH = 7.4)) for 10 min.

Step 3.4.1.7: Dilute 1 mg/mL streptavidin solution in HEPES buffer to a concentration of 0.125 mg/mL.

Step 3.4.1.8: Incubate the channel at room temperature with 20 μL of the streptavidin solution for 5 min.

Step 3.4.1.9: Add 20 μL of GUV components and incubate at room temperature for 5 min.

Step 3.4.1.10: Add 60 μL of HEPES buffer at one end of the channel and use qualitative filter paper to draw the liquid flow avoiding the introduction of gas bubbles.

Step 3.4.1.11: Once the above operations are completed, the channels should be immediately photographed at selected points, and the process should be completed within 10 min.

###### Step 3.4.2: Migrasome reconstitution assay via tether pulling [TIMING 0.5 d]

Step 3.4.2.1: The home-made chamber was first blocked using 1% BSA in HEPES buffer for 10 min.

Step 3.4.2.2: Close the chamber with mineral oil on two sides.

Step 3.4.2.3: GUVs (10 µL) were injected into the middle of the chamber by the extended tip.

Step 3.4.2.4: When the GUVs became stable in the system, a glass needle was inserted into the chamber and attached to the target GUV.

Step 3.4.2.5: The glass needle was pulled to one side to deform the GUV.

##### Step 4: Imaging of migrasomes by microscopy

**Figure 8:**
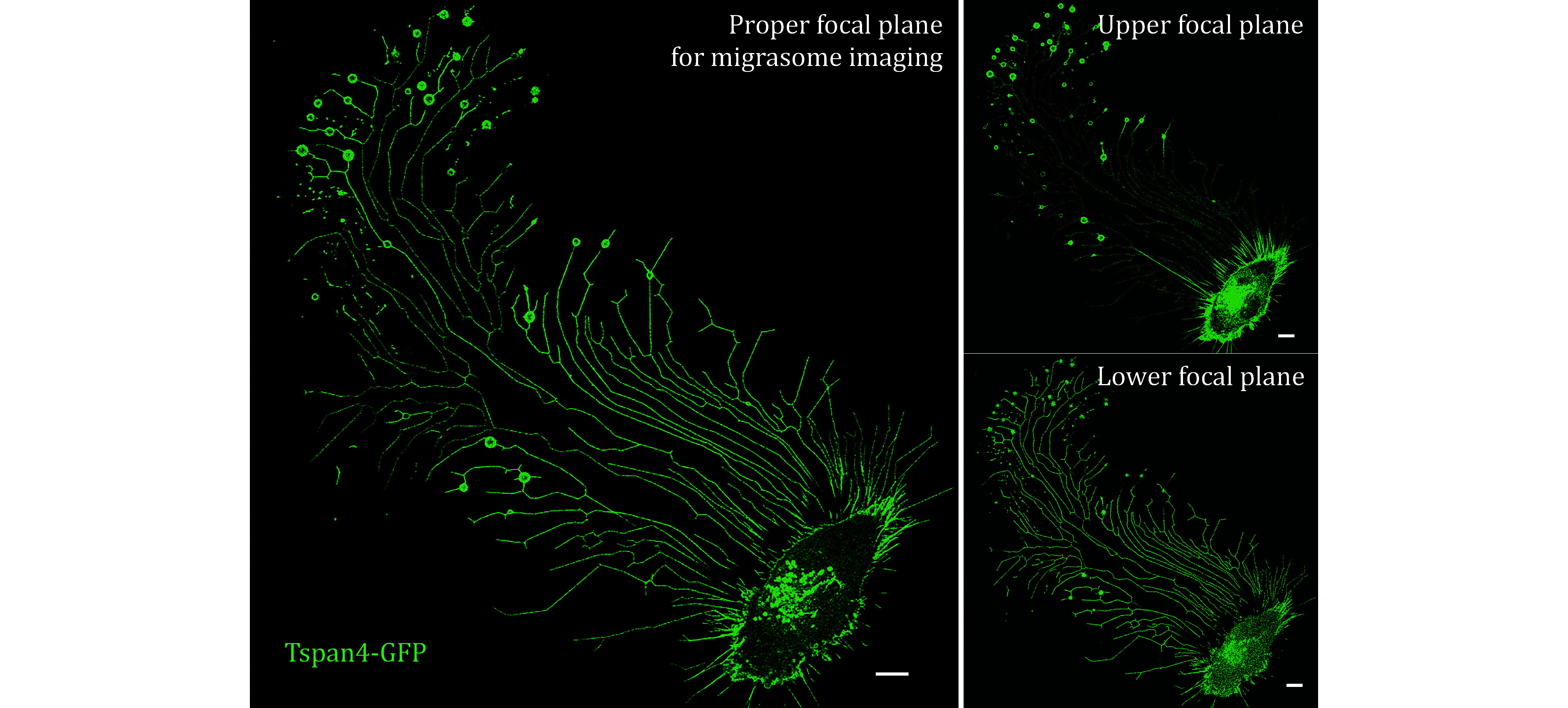
Migrasome images of Tetraspanin4-GFP over-expressed L929 cells were collected by confocal microscopy at a proper focal plane or improper (Upper or Lower) focal plane. Scale bar, 10 μm

Imaging is the final step in observing migrasomes. Confocal microscopy is the most used method for clear migrasome observation. For higher spatial and temporal resolution, spinning disc confocal microscopy, structured illumination microscopy (SIM), total internal reflection fluorescence microscopy (TIRF), and stochastic optical reconstruction microscopy (STORM) are powerful tools that can potentially be applied for enhanced migrasome visualization. The key to obtaining high-quality migrasome images is ensuring a precise and stable imaging focal plane as shown in Fig. 8, especially during time-lapse imaging.

###### Step 4.1: Imaging of cell migrasomes [TIMING 0.5 d]

####### Step 4.1.1: For living-cell samples

Step 4.1.1.1: Run the living-cell culture system equipped with microscopy ahead to ensure the system reaches 37 °C and 5% CO_2_.

Step 4.1.1.2: Preset 60× objective or 100× objective for precise capture of migrasomes.

Step 4.1.1.3: Put the cell samples onto microscopy and find the proper focal plane, usually approximately to the cell bottom at which the retraction fibers and migrasomes could be captured at the same time.

Step 4.1.1.4: Time-lapse imaging for the migrasome biogenesis process is suggested to perform at 4 min intervals and at least 6 h with relatively lower laser intensity to avoid cell damage.

For fixed cell samples, you could follow Steps 4.1.1.2 and 4.1.1.3 to take images.

###### Step 4.2: Imaging of in vitro reconstituted migrasomes [TIMING 0.5 d]

Step 4.2.1: Find or make a proper adaptor for reconstituted samples. For reconstitution by tether pulling, you need a set of 3D micromanipulation instruments to realize real-time operation and observation.

Step 4.2.2: Preset 60× objective, 488-nm and 561-nm lasers.

**Figure 9:**
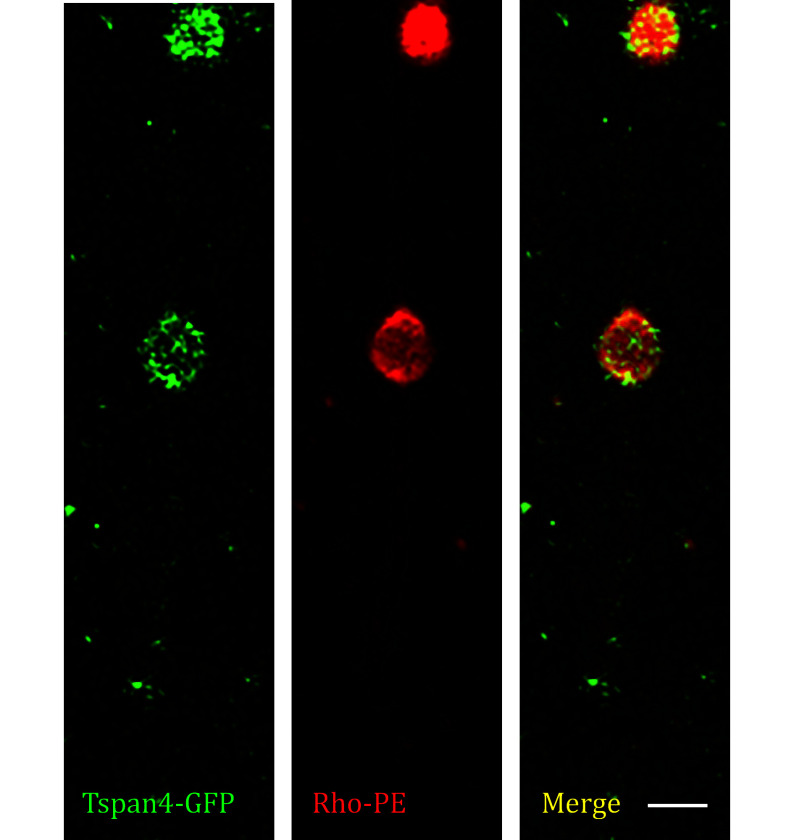
Z-stack images of *in vitro* reconstituted migrasome by flow channel, collected by confocal microscopy. Green, GFP; red, rhodamine; yellow, merge. Scale bar, 2 μm

Step 4.2.3: You may need to use Z-stack imaging with 0.2-µm step size to fully capture the reconstituted retraction fibers and migrasomes, the final images are shown in Fig. 9.

Step 4.2.4: For real-time observation of reconstitution by pulling, 512 × 512 pixel frame, line scanning mode and with no interval time lapse imaging was recommended.

##### Step 5: Statistics of migrasomes

Migrasome production occurs over time and space, which means that the number of migrasomes typically follows a certain distribution within a range. Basic statistics on migrasome numbers are necessary to draw conclusions from our observations, especially when making comparisons between groups. To ensure objectivity, images used for statistics should be taken randomly. It's recommended that the sample size include 100 cells from at least three independent repetitions.

###### Step 5.1: Migrasome counting

####### Step 5.1.1: Migrasome number per cell [TIMING 1 h per sample]

Step 5.1.1.1: Keep the standard migrasome morphology under light microscopy in mind and keep the unified principle of counting all groups of samples during one experiment.

Step 5.1.1.2: Use Image J software to open migrasome images.

Step 5.1.1.3: Count the total migrasome number of each cell and record the number one cell by one cell in case the need for double check.

Step 5.1.1.4: 100 cells from three independent repeats need to be counted.

####### Step 5.1.2: Migrasome number per tether length [TIMING 0.5 d per sample]

Step 5.1.2.1: Use Image J software to open migrasome images.

Step 5.1.2.2: Count the total migrasome number of each cell and record the number one cell by one cell.

Step 5.1.2.3: Track the retraction fibers and measure the total length of each cell.

Step 5.1.2.4: Normalize the migrasome number by the total length of retraction fiber of each cell.

####### Step 5.1.3: Reconstituted migrasome number per tether length [TIMING 0.5 d per sample]

Step 5.1.3.1: Use Image J software to open reconstituted migrasome images.

Step 5.1.3.2: Count migrasome numbers and tether length of each tether.

Step 5.1.3.3: Normalize the migrasome number by tether length.

Step 5.1.3.4: At least 30 frames of images from three independent repeats are the recommended sample size.

###### Step 5.2: Analysis for the significance of difference [TIMING 1 h per set of experiments]

Step 5.2.1: Open the original counting results in GraphPad Prism software and draw column graphs.

Step 5.2.2: When the sample size is relatively large or the data fits the normalization distribution, use an unpaired two-tail *t*-test to analyze the differences.

Step 5.2.3: If the data do not fit normalization distribution, use distribution-free methods, such as the Mann-Whitney U test.

## ADVANTAGES AND LIMITATIONS OF THIS PROTOCOL

The step-by-step procedure provides a standardized guideline for observing cell migrasomes through a microscope, which is the most efficient method for verifying the production of migrasomes in a specific cell type. High-quality labeling and imaging of migrasomes provide sufficient analysis of their characteristics and support biogenesis research. Furthermore, the *in vitro* reconstitution system for migrasomes provides a robust tool and a different perspective for deciphering migrasome formation and regulatory mechanisms. The statistical method is customized to provide a relatively convincing judgment of the impacts on migrasomes. However, this protocol provides basic instructions for migrasome studies. Depending on different research objects and personalized purposes, the protocol details may need slight adjustments to align with your specific aims. With the ongoing progress in migrasome research and the continuous advancement of techniques, the protocol for studying migrasomes needs to be expanded and updated.

## Conflict of interest

Yuwei Huang and Li Yu declare that they have no conflict of interest.
